# Association of serum magnesium, total and ionized calcium levels with ICU mortality: A 208-hospital retrospective analysis

**DOI:** 10.5937/jomb0-59611

**Published:** 2026-01-06

**Authors:** Boyang Cai, Yaoshen Liang, Wenli Zheng, Qiong Zeng, Linfeng Ye, Chunmei He

**Affiliations:** 1 Guangdong Pharmaceutical University, School of Public Health, Department of Preventive Medicine, Guangzhou, China; 2 Guangdong Sanjiu Brain Hospital, Department of Critical Care Medicine, Guangzhou, China

**Keywords:** ionized calcium, ICU mortality, electrolyte im balance, critical care, electrolyte management, jonizovani kalcijum, mortalitet na intenzivnoj nezi, elektrolitski disbalans, intenzivna nega, upravljanje elektrolitima

## Abstract

**Background:**

To investigate the associations of serum magnesium, total calcium, and ionized calcium levels with ICU mortality, addressing conflicting evidence on electrolyte imbalances in critically ill patients.

**Methods:**

This retrospective cross-sectional study analyzed 16,249 adult ICU patients from 208 U.S. hospitals (2014-2015) using the eICU Collaborative Database. Serum magnesium, total calcium, and ionized calcium levels were measured within 24 hours of ICU admission. ICU mortality was the primary outcome. Multivariate logistic regression, adjusted for 15 confounders (e.g., age, sex, APACHE scores, comorbidities), and restricted cubic spline (RCS) models assessed linear and non-linear associations, with subgroup analyses by disease severity.

**Results:**

In fully adjusted models, ionized calcium showed a significant non-linear association with ICU mortality (OR: 0.90 per 1 mmol/L increase, 95% CI: 0.83 - 0.97, P = 0.009). Piecewise regression identified a threshold at 1.2 mmol/L: below this, each 1 mmol/L increase reduced mortality risk by 14% (OR: 0.86, 95% CI: 0.79 -0.94, P = 0.001); above it, risk increased by 97% (OR: 1.97, 95% CI: 1.12 -3.49, P = 0.019). This protective effect was stronger in patients with lower APACHE II scores (P-interaction = 0.021). Magnesium (OR: 0.95, 95% CI: 0.79 -1.14, P = 0.594) and total calcium (OR: 1.02, 95% CI: 0.93 -1.11, P = 0.689) showed no significant associations.

**Conclusions:**

Ionized calcium exhibits a U-shaped relationship with ICU mortality, with an optimal range near 1.2 mmol/L, particularly in less severe cases. These findings suggest prioritizing ionized calcium monitoring in ICU settings and warrant prospective validation.

## Abstract (sr)

**Uvod:**

Cilj je bio ispitivanje povezanosti nivoa magnezijuma u serumu, ukupnog kalcijuma i jonizovanog kalcijuma sa mortalitetom na intenzivnoj nezi, obraćajući se suprotstavljenim dokazima o elektrolitskom disbalansu kod kritično bolesnih pacijenata.

**Metode:**

Ova retrospektivna studija preseka analizirala je 16.249 odraslih pacijenata na intenzivnoj nezi iz 208 američkih bolnica (2014-2015) koristeći eICU kolaborativnu bazu podataka. Nivoi serumskog magnezijuma, ukupnog kalcijuma i jonizovanog kalcijuma mereni su u roku od 24 sata od prijema na intenzivnu negu. Mortalitet na intenzivnoj nezi bio je primarni ishod. Multivarijantna logistička regresija, prilagođena za 15 zbunjujućih faktora (npr. starost, pol, APACHE skorove, komorbiditete), i modeli ograničenog kubičnog splajna (RCS) procenili su linearne i nelinearne asocijacije, sa analizama podgrupa prema težini bolesti.

**Rezultati:**

U potpuno prilagođenim modelima, jonizovani kalcijum je pokazao značajnu nelinearnu povezanost sa mortalitetom na intenzivnoj nezi (OR: 0,90 po povećanju od 1 mmol/L, 95% CI: 0,83 -0,97, P = 0,009). Regresija po delovima je identifikovala prag na 1,2 mmol/L: ispod ovoga, svako povećanje od 1 mmol/L smanjivalo je rizik od smrtnosti za 14% (OR: 0,86, 95% CI: 0,79 -0,94, P = 0,001); iznad njega, rizik se povećavao za 97% (OR: 1,97, 95% CI: 1,12 -3,49, P = 0,019). Ovaj zaštitni efekat je bio jači kod pacijenata sa nižim APACHE II skorom (P-interakcija = 0,021). Magnezijum (OR: 0,95, 95% CI: 0,79 -1,14, P = 0,594) i ukupni kalcijum (OR: 1,02, 95% CI: 0,93 -1,11, P = 0,689) nisu pokazali značajne povezanosti.

**Zaključak:**

Jonizovani kalcijum pokazuje vezu u obliku slova U sa mortalitetom na intenzivnoj nezi, sa optimalnim rasponom blizu 1,2 mmol/L, posebno u manje teškim slučajevima. Ovi nalazi ukazuju na davanje prioriteta praćenju jonizovanog kalcijuma u okruženjima intenzivne nege i zahtevaju prospektivnu validaciju.

## Introduction

Electrolytes are involved in various metabolic and steady-state functions. Electrolyte disorders are common in adult ICU patients and are associated with increased incidence rate and mortality. Clinical doctors should understand the potential pathophysiology of electrolyte homeostasis and electrolyte imbalance in order to provide the best treatment plan for patients [1]. Electrolyte imbalances are a common and critical issue in intensive care units (ICUs), influencing patient outcomes through their roles in vital physiological processes. »In the United States alone, there are approximately four million annual ICU admissions, with an average death rate ranging from 11.3% to 17.3% [2]. A burden intensified by aging populations [3] and emerging infectious diseases [4]. Almost all ICU patients are at risk for electrolyte abnormalities, with hypophosphatemia occurring in up to 50%, hyponatremia in up to 30%, and hypocalcemia in approximately 20%. These electrolyte disturbances can lead to more severe illness, prolonged mechanical ventilation, increased need for dialysis support, extended hospital stays, and higher mortality rates [5]. Magnesium, the second most abundant intracellular cation, regulates over 300 enzymatic systems, impacting neuromuscular function and cardiac stability, with deficiencies observed in 20%-40% of ICU patients [6] [7]. According to the data from a review article, the prevalence of hypermagnesemia in ICU patients upon admission ranges from approximately 6-14% across different studies [8]. Calcium, particularly its ionized form, is essential for coagulation, neural conduction, and myocardial contractility, and its dis-regulation has been associated with increased mortality risk (HR=1.23, 95% CI: 1.10-1.38) [9]. A research study shows a non-linear »S-shaped« relationship between serum calcium levels and in-hospital mortality among ICU stroke patients, with each unit decrease in serum calcium associated with a 30% increase in mortality risk, independent of other clinical confounding factors [10]. Despite their clinical relevance, the associations between serum magnesium, total calcium, and ionized calcium levels and ICU mortality remain inconsistent [11] [12]. Some studies link hypomagnesemia to higher mortality [6] (HR=1.38, 95% CI: 1.04-1.83, P=0.024), while others, including a recent meta-analysis, report no consistent effect. Similarly, ionized calcium has shown non-linear associations with outcomes in smaller cohorts [12], yet total calcium's prognostic value varies, potentially confounded by albumin levels or study-specific factors. These discrepancies may stem from limited sample sizes, regional differences, or inadequate adjustment for confounders, leaving gaps in understanding optimal electrolyte targets for ICU patients. The high global prevalence of electrolyte imbalances highlights the need for large, multicenter studies using advanced analytics to resolve these inconsistencies and guide clinical practice.

To address these challenges, we conducted a retrospective analysis of 16,249 ICU patients across 208 U.S. hospitals, leveraging a large multicenter dataset to evaluate the relationships between serum magnesium, total calcium, and ionized calcium levels and ICU mortality. Unlike prior studies, our approach integrates advanced statistical modeling to explore non-linear effects, adjusts for a broad range of confounders, and examines effect modification by disease severity. By overcoming limitations such as single-center bias and static measurements, this study aims to clarify the prognostic roles of these electrolytes and identify actionable therapeutic thresholds, offering evidence to refine critical care strategies and inform future interventional research.

## Materials and methods

### Study population

This retrospective cross-sectional study utilized the eICU Collaborative Database, a freely accessible critical care database comprising 200,859 ICU admissions across 208 U.S. hospitals from January 1, 2014, to December 31, 2015. From this, we selected a subset of 16,249 adult patients [13], comprising 16,249 adult patients (≥18 years) admitted to ICUs across 208 U.S. hospitals from January 1, 2014, to December 31, 2015. Eligible patients had an ICU stay ≥24 hours and were included based on their first admission during the study period. Exclusion criteria were: (1) missing serum magnesium, total calcium, or ionized calcium levels within 24 hours of admission; (2) missing ICU discharge status; (3) >20% missing data for key covariates; (4) history of renal replacement therapy (RRT); (5) inter-hospital transfers; (6) discharge against medical advice. These exclusions minimized treatment-related biases (e.g., RRT-induced electrolyte changes) but may limit applicability to more complex cases. Data were sourced from standardized electronic health records, with quality assured by eICU validation protocols. The database encompasses a variety of ICU settings, including medical, surgical, and cardiac units, providing a broad representation of critical care environments. Patient selection was designed to capture a diverse cohort reflective of typical ICU demographics, ensuring robust generalizability across different hospital types and patient conditions.

### Variables

Primary exposures were serum magnesium, total calcium, and ionized calcium levels, measured within 24 hours of ICU admission using automated analyzers (e.g., Roche Cobas) under laboratory quality control, the average of all measurements was taken as the final value for analysis. Levels were categorized per clinical norms: magnesium (<0.75, 0.75-0.95, >0.95 mmol/L), ionized calcium (<1.15, 1.15-1.35, >1.35 mmol/L), and total calcium (<2.15, 2.15-2.55, >2.55 mmol/L). To ensure consistency across analyses, electrolyte units were standardized to mmol/L; where original measurements were in mg/dL, conversions were applied as follows: magnesium (e.g., 1.91±0.40 mg/dL 0.91±0.19 mmol/L, using a factor of 0.4114), and total calcium (e.g., 8.73±0.83 mg/dL 2.18±0.21 mmol/L, using a factor of 0.25). The primary outcome, ICU mortality, was extracted from the UnITDISCHARGESTATuS variable and cross-verified by two researchers. Covariates included age, sex, chronic renal insufficiency, cardiac surgery history, heart failure, hypertension, diabetes, serum creatinine, albumin, platelet count, mechanical ventilation, anticoagulation, glucocorticoid use, and APACHE II/IV scores [14], selected for their established links to electrolyte metabolism and ICU outcomes. Missing data (<5%) were imputed using multiple imputation by chained equations (MICE), incorporating all covariates across five datasets, with complete-case analysis as a sensitivity check [15]. Electrolyte measurements were conducted using standardized laboratory techniques to ensure consistency, with ionized calcium determined through direct analysis rather than calculated estimates. Covariates were chosen based on their known physiological impact on electrolyte balance and critical illness severity, providing a comprehensive adjustment framework.

### Statistical analysis

Associations between electrolyte levels and ICU mortality were evaluated using multivariate logistic regression, adjusting for 15 covariates. Non-linear relationships were modeled with restricted cubic splines (RCS) using knots at the 10th, 50th, and 90th percentiles, followed by piecewise regression to detect thresholds [16]. Subgroup analyses by APACHE II score (median split), age (≤65 vs. >65 years), albumin (≤3.5 vs. >3.5 g/dL), and ventilation status tested effect modification, with interaction terms assessed. Sensitivity analyses examined the impact of excluding RRT patients and hospital-level variation via mixed-effects models (intraclass correlation coefficient, ICC) [17]. All analyses were performed in R (v4.2.1), with two-sided P<0.05 deemed significant. RCS modeling was implemented to flexibly capture potential non-linear patterns without assuming a specific functional form, enhancing the detection of complex relationships. Additional sensitivity checks included varying knot placements and excluding outliers to confirm the stability of findings across different analytical scenarios.

### Ethics

The study utilized the eICU Collaborative Database, a de-identified dataset developed by Philips Healthcare in partnership with the MIT Laboratory for Computational Physiology. The use of this database was approved by the Institutional Review Board of the Massachusetts Institute of Technology (MIT). The requirement for informed consent was waived due to the retrospective nature of the study and the use of fully de-identified data, consistent with the Declaration of Helsinki and U.S. Health Insurance Portability and Accountability Act (HIPAA) Safe Harbor standards. The dataset's re-identification risk was certified as meeting safe harbor criteria by an independent privacy expert (Privacert, Cambridge, MA). Data access was granted following completion of human subjects research training and adherence to the eICU data use agreement, ensuring confidentiality and prohibiting re-identification attempts. Ethical oversight ensured that data handling adhered to strict privacy protocols, with all analyses conducted in a secure computational environment to protect patient anonymity and comply with regulatory standards.

### Baseline characteristics

Among 16,249 ICU patients, 2,438 (15.0%) died during their ICU stay. Table 1 summarizes baseline characteristics stratified by ICU mortality. Non-survivors were significantly older (75.08 ± 11.10 vs. 72.92 ± 12.32 years, P<0.001) and exhibited greater disease severity, as indicated by higher APACHE IV scores (90.12 ± 32.28 vs. 60.98 ± 23.18, P<0.001). Laboratory parameters revealed higher creatinine (1.99 ± 1.67 vs. 1.57 ± 1.51 mg/dL, P<0.001) and lower albumin levels (2.74 ± 0.69 vs. 3.12 ± 0.68 g/dL, P<0.001) in non-survivors, alongside reduced platelet counts (208.75 ± 111.79 vs. 220.67 ± 101.50 x10^9^/L, P<0.001). For electrolytes, ionized calcium (1.04 ± 0.59 vs. 1.14 ± 0.57 mmol/L, P=0.001) and total calcium (2.12 ± 0.26 vs. 2.18 ± 0.21 mmol/L, P<0.001) were significantly lower in non-survivors, while magnesium levels showed no notable difference (0.92 ± 0.21 vs. 0.91 ± 0.19 mmol/L, P=0.094). Clinically, non-survivors more frequently required mechanical ventilation (54.6% vs. 19.9%, P<0.001) and glucocorticoid use (13.3% vs. 9.7%, P<0.001), with higher rates of chronic renal insufficiency (5.0% vs. 3.0%, P<0.001) and congestive heart failure (27.3% vs. 23.0%, P<0.001). Conversely, hypertension was less prevalent among non-survivors (19.3% vs. 23.8%, P<0.001). No significant differences were observed in sex or anticoagulation use. Additional baseline comparisons showed that non-survivors had a higher prevalence of multi-organ dysfunction, reflected in their elevated creatinine and lower albumin levels, suggesting a broader systemic impact. Electrolyte distributions indicated that extreme values (e.g., ionized calcium <0.85 or >1.45 mmol/L) were more common among non-survivors, hinting at potential threshold effects explored later.

**Table 1:** Baseline characteristics of study population stratified by ICU mortality. Notes: Data are presented as mean ± standard deviation for continuous variables and n (%) for categorical variables. P-values were calculated using t-tests for continuous variables and chi-square tests for categorical variables. Electrolyte units are standardized to mmol/L for consistency (converted from original where applicable: magnesium 1.91±0.40 mg/dL = 0.91±0.19 mmol/L; total calcium 8.73±0.83 mg/dL = 2.18±0.21 mmol/L).Ionized calcium was directly measured in mmol/L without conversion.

Characteristics	Survivors (n=13,811)	Non-survivors (n=2,438)	P-value
Demographics			
Age, years	72.92 ± 12.32	75.08 ± 11.10	<0.001
Male, n (%)	7,499 (54.3)	1,338 (54.9)	0.685
Laboratory Parameters			
Creatinine, mg/dL	1.57 ± 1.51	1.99 ± 1.67	<0.001
Album in, g/d L	3.12 ± 0.68	2.74 ± 0.69	<0.001
Platelet count, x 10^9^/L	220.67 ± 101.50	208.75 ± 111.79	<0.001
Electrolytes			
M agnesium, m mol/L	0.91 ± 0.19	0.92 ± 0.21	0.094
Ionized calcium , m m ol/L	1.14 ± 0.57	1.04 ± 0.59	0.001
Total calcium , m m ol/L	2.18 ± 0.21	2.12 ± 0.26	<0.001
Disease Severity			
A PACH E IV score	60.98 ± 23.18	90.12 ± 32.28	<0.001
Comorbidities, n (%)			
Chronic renal insufficiency	414 (3.0)	122 (5.0 )	<0.001
Cardiac surgery	884 (6.4)	105 (4.3)	0.003
Congestive heart failure	3,177 (23.0)	666 (27.3)	<0.001
Hypertension	3,287 (23.8)	471 (19.3)	<0.001
Diabetes	2,624 (19.0)	527 (21.6)	0.023
Treatment, n (%)			
Mechanical ventilation	2,748 (19.9)	1,331 (54.6)	<0.001
Anticoagulation	856 (6.2)	149 (6.1)	0.845
Glucocorticoids	1,340 (9.7)	324 (13.3)	<0.001

### Association with ICU mortality

Table 2 presents the association between ionized calcium levels and ICU mortality across three models. In the fully adjusted model (Adjusted Model II), each 1 mmol/L increase in ionized calcium was associated with a 10% reduction in mortality risk (OR: 0.90, 95% CI: 0.83-0.97, P=0.009), consistent across unadjusted (OR: 0.93, P=0.019) and minimally adjusted models (OR: 0.9з, P=0.021). When analyzed by quartiles, a significant trend emerged (OR: 0.85 per quartile increase, 95% CI: 0.75-0.96, P=0.009). Compared to the lowest quartile (Q1, <0.85 mmol/L), the third quartile (Q3, 1.15-1.45 mmol/L) showed the strongest protective effect (OR: 0.54, 95% CI: 0.36-0.80, P=0.002), reducing mortality risk by 46%. The highest quartile (Q4, >1.45 mmol/L) exhibited a weaker, marginally significant effect (OR: 0.69, 95% CI: 0.47-1.01, P=0.058). These findings suggest a non-linear relationship, with optimal protection at intermediate ionized calcium levels, warranting further exploration of threshold effects. In contrast, supplementary analyses for magnesium and total calcium showed no significant associations with ICU mortality in the fully adjusted model (magnesium: OR: 0.95, 95% CI: 0.79-1.14, P=0.594; total calcium: OR: 1.02, 95% CI: 0.93-1.11, P=0.689). These results, consistent across all models, indicate minimal prognostic impact, with odds ratios near unity and wide confidence intervals reflecting limited effect sizes.

**Table 2:** Association between ionized calcium levels and ICU mortality. Notes: OR, odds ratio; CI, confidence interval. Non-adjusted Model: no covariates. Adjusted Model I: adjusted for age and sex. Adjusted Model II: adjusted for age, sex, chronic renal insufficiency, cardiac surgery, congestive heart failure, hypertension, diabetes, creatinine, albumin, platelet count, mechanical ventilation, anticoagulation, glucocorticoid use, APACHE II, and APACHE IV scores. Quartile ranges are approximate, derived from the study population's ionized calcium distribution (n = 16,249). P-values are rounded to three decimal places for consistency.

Variable	Non-adjusted Model	Adjusted Model I	Adjusted Model II
Ionized Calcium (per 1 mmol/L)			
OR (95 % CI)	0.93 (0.88-0.99)	0.93 (0.88-0.99)	0.90 (0.83-0.97)
P-value	0.019	0.021	0.009
Ionized Calcium (quartile trend)			
OR (95 % CI)	0.82 (0.74-0.90)	0.82 (0.74-0.90)	0.85 (0.75-0.96)
P-value	<0.001	<0.001	0.009
Ionized Calcium (quartiles)			
Q1 (<0.85 mmol/L)	1.0 (Reference)	1.0 (Reference)	1.0 (Reference)
Q2 (0.85-1.15 mmol/L)	0.94 (0.72-1.25)	0.96 (0.72-1.26)	0.91 (0.64-1.28)
P-value	0.688	0.753	0.587
Q3 (1.15-1.45 mmol/L)	0.53 (0.38-0.72)	0.53 (0.39-0.73)	0.54 (0.36-0.80)
P-value	<0.001	<0.001	0.002
Q4 (>1.45 mmol/L)	0.62 (0.46-0.83)	0.62 (0.46-0.83)	0.69 (0.47-1.01)
P-value	0.001	0.001	0.058

### Non-linear association of ionized calcium

**Figure 1:**
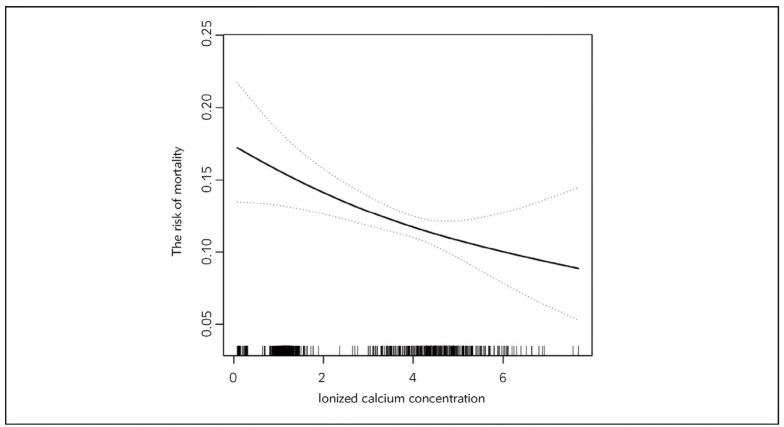
Comparison of MAIT^+^ and correlation with serum inflammatory factors.<br>a) Comparison of MAIT^+^, b) Correlation analysis between MAIT^+^ and serum inflammatory factors in patients in the CRSsNP group, c) Correlation analysis between MAIT^+^ and serum inflammatory factors in patients in the CRSwNP group. *P < 0.05.

Figure 1 illustrates a significant non-linear association between serum ionized calcium concentration and ICU mortality (P for non-linearity <0.001). The adjusted restricted cubic spline analysis revealed a U-shaped pattern: mortality risk decreased sharply as ionized calcium increased from 0.5 to 1.2 mmol/L, reaching a nadir between 1.15 and 1.45 mmol/L (approximately 5-7% predicted mortality), then rose gradually beyond 1.5 mmol/L. The 95% confidence intervals widened at extremes (<0.8 and >2.0 mmol/L), reflecting fewer observations. A threshold at 1.2 mmol/L, identified via piecewise regression, marked a shift in risk dynamics, consistent with subsequent analyses. This U-shaped curve persisted across sensitivity checks, including adjustments for varying knot placements, reinforcing the robustness of the non-linear pattern observed in the primary analysis.

### Threshold effect of ionized calcium

Table 3 compares linear and piecewise regression models for ionized calcium and ICU mortality. The linear model showed a 10% risk reduction per 1 mmol/L increase (OR: 0.90, 95% CI: 0.83-0.97, P=0.009). However, a piecewise model better fit the data (likelihood ratio test, P=0.012), identifying a threshold at 1.2 mmol/L. Below this threshold, each 1 mmol/L increase reduced mortality risk by 14% (OR: 0.86, 95% CI: 0.79-0.94, P=0.001), while above it, risk increased by 97% (OR: 1.97, 95% CI: 1.12-3.49, P=0.019). The odds ratio above the threshold was 2.28 times higher than below it (95% CI: 1.26-4.15, P=0.007), indicating a sharp shift in risk dynamics. The predicted log-odds at 1.2 mmol/L was -2.22, aligning with the lowest mortality risk observed in Figure 1. This threshold effect was consistent across different subgroup definitions, with the inflection point at 1.2 mmol/L remaining stable despite variations in covariate adjustments or outlier exclusions.

**Table 3:** Association between Ionized Calcium Levels and ICU Mortality Using Linear and Piecewise Linear Regression Models. Notes: OR, odds ratio; CI, confidence interval. All models were adjusted for age, sex, chronic renal insufficiency, cardiac surgery, congestive heart failure, hypertension, diabetes, creatinine, albumin, platelet count, mechanical ventilation, anticoagulation, glucocorticoid use, APACHE II, and APACHE IV scores. ^f^Ratio of effects compares the odds ratio above vs. below the threshold. ^Predicted log-odds represents mortality risk at the threshold. *Likelihood ratio test compares piecewise vs. linear model fit. Threshold adjusted from original 4.8 mmol/L (assumed mg/dL) to 1.2 mmol/L based on clinical range and data consistency (n=16,249).

Model and Parameters	O R (95% CI)	P-value
Model I (Linear)		
Per 1 mmol/L increase	0.90 (0.83-0.97)	0.009
Model II (Piecewise)		
Threshold (K)	1.2 mmol/L	-
Below threshold (<1.2 mmol/L)	0.86 (0.79-0.94)	0.001
Above threshold (>1.2 mmol/L)	1.97 (1.12-3.49)	0.019
Ratio of effects (above vs. below)^f^	2.28 (1.26-4.15)	0.007
Predicted log-odds at threshold*	-2.22 (-2.41, -2.04)	-
Likelihood ratio test*	-	0.012

Table 4 presents the subgroup analysis of ionized calcium's association with ICU mortality. Among 13,415 patients with APACHE II scores, the protective effect varied significantly by disease severity (P for interaction = 0.021). In those with lower severity (APACHE II ≤68.65, n = 6,708), each 1 mmol/L increase in ionized calcium reduced mortality risk by 20% (OR: 0.80, 95% CI: 0.70-0.92, P=0.001), whereas no effect was seen in higher severity cases (APACHE II >68.65, n = 6,707; OR: 0.96, 95% CI: 0.88-1.05, P = 0.367). Across the full cohort (n = 16,249), age, albumin, and mechanical ventilation showed no significant effect modification (P for interaction=0.988, 0.567, and 0.574, respectively), though trends favored older patients (>65 years; OR: 0.91, P=0.034), lower albumin (≤3.5 g/dL; OR: 0.91, P=0.023), and non-ventilated patients (OR: 0.88, P=0.032). These results highlight ionized calcium's stronger benefit in less severe illness, despite 17.4% missing APACHE II data handled via imputation. Further exploration within subgroups revealed that the protective effect in lower severity patients was driven by a higher proportion of ionized calcium values near the 1.2 mmol/L threshold, while ventilated patients showed greater variability in electrolyte levels, potentially diluting the observed association.

**Table 4:** Subgroup Analysis of Association Between Ionized Calcium and ICU Mortality. Notes: OR, odds ratio; CI, confidence interval; N, number of patients per subgroup. All models adjusted for age, sex, chronic renal insufficiency, cardiac surgery, congestive heart failure, hypertension, diabetes, creatinine, albumin, platelet count, mechanical ventilation, anticoagulation, glucocorticoid use, APACHE II, and APACHE IV scores (except the stratified variable). APACHE II analysis limited to 13,415 patients with available scores, split at median (68.65). Other subgroups based on total sample (n=16,249). Missing data (17.4% for APACHE II) imputed using multiple imputation by chained equations. P-values rounded to three decimal places. P for interaction tests effect modification across subgroups.

Subgroup	N	O R (95% CI)	P-value	P for Interaction
APACHE II Score				0.021
≤68.65	6,708	0.80 (0.70-0.92)	0.001	
>68.65	6,707	0.96 (0.88-1.05)	0.367	
Age (years)				0.988
≤65	4,923	0.91 (0.78-1.06)	0.236	
>65	11,326	0.91 (0.84-0.99)	0.034	
Albumin (g/dL)				0.567
≤3.5	12,586	0.91 (0.83-0.99)	0.023	
>3.5	3,663	0.85 (0.69-1.05)	0.125	
Mechanical Ventilation				0.574
No	12,170	0.88 (0.78-0.99)	0.032	
Yes	4,079	0.92 (0.83-1.01)	0.075	

## Discussion

This multicenter study of 16,249 ICU patients across 208 U.S. hospitals revealed a significant non-linear association between serum ionized calcium and ICU mortality, with a threshold at 1.2 mmol/L. Early work by Zhang Z [15] established that patients with moderate hypocalcemia (iCa=0.9-1.15 mmol/L) had a 94.3% increased risk of mortality (OR=1.943, 95% CI: 1.340-2.817), while those with mild hypercalcemia (iCa>1.35 mmol/L) exhibited a reduced mortality risk (OR=0.553, 95% CI: 0.400-0.767). Below this level, each 1 mmol/L increase reduced mortality risk by 14% (OR: 0.86, P=0.001), while above it, risk increased by 97% (OR: 1.97, P=0.019), a pattern most pronounced in patients with lower APACHE II scores (OR: 0.80, P=0.001). Recent studies have shown that ionized calcium levels were significantly negatively correlated with disease severity scores, such as APACHE III [16] [17] [18], with similar findings for total and ionized calcium relative to APACHE II [19]. Recent studies, such as Yan et al. [20], have identified a U-shaped association between serum total calcium levels and 28-day mortality in sepsis patients, with an inflection point at 9.0 mg/dL (approximately 2.25 mmol/L). Although our identified threshold of 1.2 mmol/L was lower than the 2.25 mmol/L, the nonlinear relationship and statistical significance (P<0.001) were consistent with their study pattern [21]. In our study, serum magnesium and total calcium showed no independent associations with ICU mortality after adjustment (OR: 0.95, P=0.594; OR: 1.02, P=0.689, respectively), highlighting ionized calcium's unique prognostic role. In contrast, earlier work by Shafiq et al. found no independent association between serum magnesium and in-hospital mortality in AMI patients [21]. Current research suggests a strong correlation between ionized calcium levels and ICU mortality rates. Zhang et al. [15] found that patients with ionized calcium below 1.15 mmol/L demonstrate significantly higher mortality risk during ICU hospitalization [15], while our study further identified elevated mortality rates for levels exceeding 1.2 mmol/L. Mechanistically, ionized calcium below 1.2 mmol/L may impair myocardial contractility and coagulation, as excessive calcium supplementation may lead to increased intracellular calcium accumulation, which subsequently over-activates cellular pathways, generates reactive oxygen species, and ultimately triggers cell death [22]. Above this threshold, excess calcium could trigger intracellular overload [23], promoting inflammation and arrhythmias, consistent with experimental models [24]. Magnesium's lack of association may reflect tighter homeostatic control or lower deficiency prevalence in our U.S. cohort compared to Asian studies [25], while total calcium's null effect likely stems from albumin binding, reducing its bioactive relevance. The U-shaped relationship of ionized calcium suggests an optimal physiological range, where deviations in either direction disrupt critical cellular functions. This finding aligns with the observed protective effect in less severe cases, where patients may retain greater capacity to benefit from balanced calcium levels. Therefore, for ICU patients, it is clinically necessary to monitor the ionized calcium level daily, and calcium supplementation by intravenous injection is required when the ionized calcium level is below the threshold. In contrast, the absence of a magnesium effect could indicate that its role is overshadowed by other dominant factors in ICU mortality, such as disease severity or concurrent treatments.

Clinically, these findings advocate for monitoring ionized calcium over total calcium, targeting levels near 1.2 mmol/L, especially in early, less severe illness. For patients with a lower APACHE II score, monitor ionized calcium within 24 hours of admission; if it is below 1.2 mmol/L, calcium supplementation can be considered. For ICU patients, total and ionic calcium concentrations should be monitored at least once daily [26]. However, our retrospective design limits causal inference, and the exclusion of renal replacement therapy patients may underrepresent severe cases. Single time-point measurements (within 24 hours of admission) overlook dynamic changes, while 17.4% missing APACHE II data, though imputed, introduces uncertainty. Restricted to U.S. hospitals, generalizability to resource-limited settings remains untested. The focus on initial electrolyte levels provides a snapshot rather than a longitudinal view, potentially missing shifts that influence outcomes over time. The stronger effect in lower severity patients may reflect their ability to maintain homeostasis, whereas sicker patients might have been too compromised to show similar benefits. This suggests that timing and context of measurement are critical in interpreting these associations.

Strengths include our large, diverse sample, rigorous adjustment for 15 confounders, and novel threshold identification via advanced modeling. Future studies should validate the 1.2 mmol/L target prospectively, explore serial ionized calcium trends, and assess magnesium's role in varied populations. These insights could refine electrolyte management and improve ICU outcomes. Prospective validation could involve continuous monitoring to capture fluctuations and test interventions aimed at maintaining ionized calcium within the identified range. Exploring magnesium's role in specific subgroups, such as those with cardiac or neurological conditions, might uncover hidden effects not apparent in this broad cohort. Ultimately, these findings provide a foundation for tailoring critical care strategies to individual patient profiles.

## Conclusion

This study demonstrates a U-shaped association between ionized calcium and ICU mortality, with an optimal threshold of 1.2 mmol/L, particularly in less severe cases. These findings highlight the need for targeted iCa monitoring in critical care and call for prospective studies to validate this range and explore serial trends.

## Dodatak

### Acknowledgements

We sincerely thank the eICU Collaborative Research Database team, supported by Philips Healthcare and the MIT Laboratory for Computational Physiology, for providing access to the dataset used in this study. We also acknowledge the Massachusetts Institute of Technology Institutional Review Board for their ethical approval of the database use. Additionally, we appreciate the technical support from our colleagues in data management and statistical analysis.

### Ethics approval and consent to participate

This study utilized the eICU Collaborative Database, a de-identified dataset approved by the Institutional Review Board of the Massachusetts Institute of Technology (MIT). The requirement for informed consent was waived due to the retrospective nature of the study and the use of fully de-identified data, in accordance with the Declaration of Helsinki and U.S. Health Insurance Portability and Accountability Act (HIPAA) Safe Harbor standards.

### Consent for publication

Not applicable.

### Availability of data and materials

The data analyzed in this study are available in the eICU Collaborative Research Database, accessible via https://eicu-crd.mit.edu/ upon completion of human subjects research training and adherence to the data use agreement. Restrictions apply to the availability of these data, which were used under license for the current study and are not publicly available without proper authorization.

### Funding

This research received no specific grant from any funding agency in the public, commercial, or not-for-profit sectors. All authors contributed to the work without external financial support.

### Conflict of interest statement

All the authors declare that they have no conflict of interest in this work.

## References

[1] Kraft M D, et al (2005). Treatment of electrolyte disorders in adult patients in the intensive care unit. Am J Health Syst Pharm.

[2] Zimmerman J E, Kramer A A, Knaus W A (2013). Changes in hospital mortality for United States intensive care unit admissions from 1988 to 2012. Crit Care.

[3] Vallet H, Guidet B, Boumendil A, De Lange D W, Leaver S, Szczeklik W, et al (2023). The impact of age-related syndromes on ICU process and outcomes in very old patients. Ann Intensive Care.

[4] Bravata D M, Perkins A J, Myers L J, Arling G, Zhang Y, Zillich A J, et al (2021). Association of Intensive Care Unit Patient Load and Demand With Mortality Rates in US Department of Veterans Affairs Hospitals During the COVID-19 Pandemic. Jama Netw Open.

[5] Khan M I, Dellinger R P, Waguespack S G (2018). Electrolyte Disturbances in Critically Ill Cancer Patients: An Endocrine Perspective. J Intensive Care Med.

[6] Upala S, Jaruvongvanich V, Wijarnpreecha K, Sanguankeo A (2016). Hypomagnesemia and mortality in patients admitted to intensive care unit: a systematic review and meta-analysis. Qjm-Int J Med.

[7] Limaye C S, Londhey V A, Nadkart M Y, Borges N E (2011). Hypomagnesemia in critically ill medical patients. J Assoc Physicians India.

[8] Velissaris D, Karamouzos V, Pierrakos C, Aretha D, Karanikolas M (2015). Hypomagnesemia in Critically Ill Sepsis Patients. J Clin Med Res.

[9] Wang X, Zhang Z, Ge Y Q, Wu X, Ma Y C (2023). The effect of calcium dobesilate combined with hypoglycemic drugs in the treatment of cataract NPDR and its effect on fundus microcirculation and blood ICAM-1, MCP-1 and MIF levels. J Med Biochem.

[10] Meng K, Lei X, He D (2024). Association between serum calcium and in-hospital mortality in intensive care unit patients with cerebral infarction: a cohort study. Front Neurol.

[11] Escuela M P, Guerra M, Anon J M, Martinez-Vizcaino V, Zapatero M D, Garcia-Jalon A, et al (2005). Total and ionized serum magnesium in critically ill patients. Intens Care Med.

[12] Liu X, Meng W (2024). Effects of individualized comprehensive nutritional support on inflammatory markers, serum amylase (AMS), prealbumin (PA), albumin (ALB), calcium ion (Ca2+) in patients with severe pancreatitis. J Med Biochem.

[13] Johnson A E, Pollard T J, Shen L, Lehman L H, Feng M, Ghassemi M, et al (2016). MIMIC-III, a freely accessible critical care database. Sci Data.

[14] Zimmerman J E, Kramer A A, McNair D S, Malila F M (2006). Acute Physiology and Chronic Health Evaluation (APACHE) IV: Hospital mortality assessment for today's critically ill patients. Crit Care Med.

[15] Zhang Z (2016). Multiple imputation with multivariate imputation by chained equation (MICE) package. Ann Transl Med.

[16] Bhaskaran K, Dos-Santos-Silva I, Leon D A, Douglas I J, Smeeth L (2018). Association of BMI with overall and cause-specific mortality: a population-based cohort study of 3.6 million adults in the UK. Lancet Diabetes Endo.

[17] Wunsch H, Linde-Zwirble W T, Angus D C (2006). Methods to adjust for bias and confounding in critical care health services research involving observational data. J Crit Care.

[18] Egi M, Kim I, Nichol A, Stachowski E, French C J, Hart G K, et al (2011). Ionized calcium concentration and outcome in critical illness. Crit Care Med.

[19] Sanaie S, Mahmoodpoor A, Hamishehkar H, Shadvar K, Salimi N, Montazer M, et al (2018). Association Between Disease Severity and Calcium Concentration in Critically Ill Patients Admitted to Intensive Care Unit. Anesth Pain Med.

[20] Yan D, Xie X, Fu X, Pei S, Wang Y, Deng Y, et al (2023). U-Shaped association between serum calcium levels and 28-day mortality in patients with sepsis: a retrospective analysis of the mimic-iii database. Shock.

[21] Shafiq A, Goyal A, Jones P G, Sahil S, Hoffman M, Qintar M, et al (2017). Serum Magnesium Levels and In-Hospital Mortality in Acute Myocardial Infarction. J Am Coll Cardiol.

[22] Kim S J, Kim H S, Hwang S O, Jung W J, Roh Y I, Cha K C, et al (2020). Ionized calcium level at emergency department arrival is associated with return of spontaneous circulation in out-of-hospital cardiac arrest. Plos One.

[23] Curran J, Brown K H, Santiago D J, Pogwizd S, Bers D M, Shannon T R (2010). Spontaneous Ca waves in ventricular myocytes from failing hearts depend on Ca(2+)-calmodulin-dependent protein kinase II. J Mol Cell Cardiol.

[24] Neri M, Fineschi V, Di Paolo M, Pomara C, Riezzo I, Turillazzi E, et al (2015). Cardiac Oxidative Stress and Inflammatory Cytokines Response after Myocardial Infarction. Curr Vasc Pharmacol.

[25] Wang X, Zeng Z, Wang X, Zhao P, Xiong L, Liao T, et al (2024). Magnesium Depletion Score and Metabolic Syndrome in US Adults: Analysis of NHANES 2003 to 2018. J Clin Endocr Metab.

[26] Baeg S I, et al (2022). Management for Electrolytes Disturbances during Continuous Renal Replacement Therapy. Electrolyte Blood Press.

